# Age, gender and household infrastructural inequality in COVID-19: Contextual analysis of Mamelodi

**DOI:** 10.4102/safp.v66i1.5924

**Published:** 2024-07-22

**Authors:** Simon M. Marcus, Caitlin V. Gardiner

**Affiliations:** 1 Department of Family Medicine, Faculty of Health Science, University of the Witwatersrand, Johannesburg, South Africa; 2 Department of Global Health and Social Medicine, King’s College, London, United Kingdom; 3 SAMRC/Wits Developmental Pathways for Health Research Unity, Department of Paediatrics, School of Clinical Medicine, University of the Witwatersrand, Johannesburg, South Africa

**Keywords:** social determinants of health, LMICs, urban health, health inequalities, vulnerable populations, health policy

## Abstract

**Background:**

Age, gender and household infrastructure are important social determinants affecting health inequalities. This study aims to assess the ways that age and gender of the household head and household infrastructure intersect to create relative advantage and disadvantage in COVID-19 vulnerability.

**Methods:**

Using household primary care survey data from Mamelodi, Gauteng, headed households were sorted into three risk categories for each of the relevant infrastructural determinants of COVID-19. Bivariate ordinal logistic regression was used to determine the odds of households falling into each risk category. The proportion of high-risk (HR) categories and dwelling types was also calculated.

**Results:**

Households headed by someone ≥ 65 years were less likely to be in all HR categories and more frequently had formal houses. Male-head households were more likely to be HR for water, sanitation and hygiene infrastructure and indoor pollution; however, female-headed households (FHHs) were at higher risk for crowding. In Mamelodi, households headed by ≥ 65 years olds were relatively infrastructurally protected, likely because of pro-equity housing policy, as were FHHs, except for crowding. The care load on FHHs results in their infrastructural protection benefiting more community members, while simultaneously incurring risk.

**Conclusion:**

Infrastructural support based on the household head’s age and gender could improve targeting and the effectiveness of health interventions. These results demonstrate the importance of a contextual understanding of gender and age inequalities and tailoring public health support based on this understanding.

**Contribution:**

This research describes patterns of health-related infrastructural inequality, identifies ways to improve health interventions, and demonstrates the importance of equity-focused policy in an African context.

## Introduction

The COVID-19 pandemic has emphasised the unequal distribution of the burden of disease with infection spread, morbidity, and mortality often following, and exacerbating, lines of social inequality.^1^ While some studies have illuminated inequalities and the intersections of social and contextual factors, early COVID-19 research often essentialised and homogenised social factors and contexts. Low- and middle-income countries (LMICs), including many African countries such as South Africa, were frequently ascribed blanket disadvantage,^2^ with limited investigation into the factors that create relative advantage and disadvantage within and between LMIC contexts. This resulted in ‘one-size-fits-all’ mitigation and suppression strategies of limited efficacy.^2^ Such homogenisation also occurred along the sociodemographic categories of age and gender.

Age has been emphasised as a significant indicator of COVID-19 vulnerability. Being 65 years or older (≥ 65) was found to be an independent risk factor for severe COVID-19 morbidity and mortality,^3^ and rationalised policy that, for example, prioritised the elderly in vaccine administration. In South Africa, however, there has been a much larger proportion of under-65 years (< 65) mortality compared to high-income countries (HICs),^4^ with the high prevalence of HIV in people < 65 years being a potential contributing factor.^5^ This indicates that the relative disadvantage of old age is not as great as in other contexts, and raised the question of whether vaccine prioritisation based primarily on age was the most appropriate policy in this context.^4^

Gender is another key risk factor emphasised in COVID-19 vulnerability. While the authors recognise the distinction between sex and gender and the pluralities of each,^6^ the terms are used interchangeably here and categorised dichotomously as ‘male or man’ and ‘woman or female’. This is because the secondary data upon which this study draw, and the South African national statistics on COVID-19,^7^ use this characterisation. Research has found men to be at greater risk of severe and fatal COVID-19,^3^ with immunological and physiological differences hypothesised to be the cause.^8^ The risk increases with age, as men aged 60–69 years have a 2.6 fold increased risk of mortality compared to similarly aged women.^9^ In South Africa, there was initially a similar gendered pattern for COVID-19; however, by the end of 2022, more South African women had been infected by, hospitalised because of, and died from COVID-19 than men.^10^ This suggests the presence of other contextual factors influencing relative gendered advantage and disadvantage in COVID-19 vulnerability. These divergences from the expected narrative of vulnerability emphasise the importance of contextual analyses of the social determinants of COVID-19. Such analyses are further useful in accounting for variations within and between regional and local contexts and planning locally appropriate health and social policy.

Household infrastructure is one of the most important social determinants of COVID-19.^11^ Inequalities in household infrastructure, which also occur along age and gendered lines, and their specificity in African city contexts have been largely unaccounted for in COVID-19 responses.^12^ This is a substantial oversight, as inequality in access to certain infrastructures has a significant effect on COVID-19 transmission, morbidity, and mortality patterns.^11^ Household infrastructural determinants of COVID-19 particularly relevant in LMICs include: water, sanitation, and hygiene (WASH), specifically water source and toilet facilities; indoor air pollution; and crowding.^13^

Household water access plays a significant role in infectious diseases generally and is vitally important in COVID-19 as hand hygiene plays a crucial role in severe acute respiratory syndrome coronavirus 2 (SARS-CoV-2) spread.^14^ Thirty per cent of the global population, however, lacks access to household water and soap,^15^ limiting their ability to follow core COVID-19 mitigation strategies. While South Africa has made strides in increasing access to clean water, only 64% have reliable and safe water supply services.^16^

Household toilets are an important WASH infrastructure influencing the spread and management of COVID-19. Toilets are high contact areas often used by multiple people from multiple households in low-income settings.^17^ These community toilets also frequently lack handwashing facilities and are less likely to be regularly cleaned.^18^ Along with SARS-CoV-2’s potential faecal–oral mechanism of spread,^19^ it makes toilet access and its associated hygiene an important factor in COVID-19 vulnerability. In South Africa, data from 2017 show that 21.6% of households reported inadequate hygiene facilities and 17.9% reported having no water to wash their hands after toilet use.^20^

Household indoor pollution from an energy source is another significant infrastructural determinant influencing COVID-19 vulnerability, particularly in LMICs. Air pollution, both indoor and outdoor, creates higher levels of particulate matter associated with increased infection and mortality rates.^21^ Particulate matter concentrations are also significantly higher when burning biomass fuels indoors.^22^ In South Africa, nearly 10% of households primarily use biomass fuels,^23^ and many more use it as a secondary source even when electricity is accessible.^24^ This COVID-19 vulnerability is compounded when combined with household crowding.

Crowding exists where the number of people in a household exceeds the available space.^25^ It is a critical determinant of COVID-19 vulnerability as it increases the risk of aerosol and droplet spread, reduces one’s ability to physically distance or self-isolate, and can increase morbidity and mortality via a progressive dose–response relationship.^26^ Poorer households tend to be disproportionately affected, especially in impoverished urban settlements with high population densities.^27^ This is true in South Africa, where a 2016 study^28^ found overcrowding in about 57% of households in two low-income Johannesburg suburbs.

A useful way to investigate how the intersections of age, gender, and household infrastructure impact relative COVID-19 vulnerability is to look at these factors in relation to the household-head. Household-headship typically refers to the household decision-maker, and the ‘principal person “to whom individuals are linked and their relationships coded”’ (p. 651).^29^ The demographics of the household-head, particularly gender and age, have been found to be an important indicator of access to various resources.^29^ The loss of the household head to COVID-19 can thus have a significant negative impact on the other household members. In South Africa, the position of household-head has typically been dominated by men, as property is commonly passed to a male relatives.^30^ This results in the social reproduction of housing infrastructural inequalities.

The importance of age and gender of the household-head has been recognised in South African infrastructural policy for almost three decades through the Reconstruction and Development Programme (RDP). This programme has been implemented by post-Apartheid governments to try and meet their constitutional obligation to provide adequate housing.^31^ It provides state-subsidised housing, commonly referred to as ‘RDP houses’,^30^ through a system of prioritised allocation targeting the most vulnerable. Here, women and the elderly are explicitly mentioned because of their increased risk of poverty and eviction, vulnerability to violence, and care needs for children and relatives.^32^ The impact of this prioritisation by gender and age, is, however, not clear. While 56% of all RDP housings were allocated to women by 2014,^33^ data that are more current or that focus on age are not readily available. Furthermore, despite government investment in housing, mismanagement combined with high demand has led to severe backlogs, resulting in the expansion of both formal and informal settlements in many urban areas.^31^

One such area is Mamelodi, a South African urban township, which exemplifies infrastructural and demographic heterogeneity. Mamelodi covers an area of about 45 km^2^ in the east of the City of Tshwane Municipality and has a population of about 334 577 people in approximately 110 703 households.^13^ Since democracy in 1994, Mamelodi has rapidly expanded because of its relative proximity to economic opportunities. Expansion has occurred through formal housing, driven by both private investment and RDP housing, as well as informal dwellings, creating high levels of infrastructural inequality in close proximity.^13^

Age, gender, and household infrastructure are all critically important in determining COVID-19 vulnerability, both independently and at their intersections. The aim of this study is to describe the distribution of household infrastructural vulnerability to COVID-19 by analysing its relationship to the age and gender of the household head, in Mamelodi. Understanding how the household infrastructural determinants of COVID-19 are distributed and what factors contribute to infrastructural inequality is essential in identifying areas for targeted support, especially in dense urban areas. It can also improve the policy that minimises household vulnerability to current and future diseases. Through this analysis, this study will provide insight into COVID-19 infrastructural inequality using the salient social determinants of age and gender, identify areas for targeted interventions, and demonstrate the importance of context and equity in policy planning.

## Research methods and design

This was a cross-sectional descriptive study using secondary data from AitaHealth^TM^, a mobile community healthcare management application developed by the University of Pretoria’s Department of Family Medicine and Mezzanineware (Vodacom).

### Data

The secondary anonymised data came from the 2018–2019 household registration survey for community-orientated primary care, covering 13 985 households in Mamelodi. It was collected by community healthcare workers and used by the Gauteng Department of Health and the University of Pretoria as part of ongoing primary health care assessments, service delivery, and research. All data were collected and analysed at a household level, with a household defined as an individual or a group of people who live together at least four nights a week and share essential resources.^34^

### Household infrastructural determinants of COVID-19

The infrastructural determinants analysed in this study were water source (indicators of the availability of water, sanitation, and hygiene infrastructure), toilet exposure, indoor pollution (the risk of air pollution from the household’s energy source), and crowding (the household’s person-to-room ratio). The choice of determinants was based on their strong links to COVID-19 vulnerability,^32^ and their availability in the data.

Households were stratified into low-risk (LR), medium-risk (MR), and high-risk (HR) groups for each of the four infrastructural determinants, as described in Table 1. Risk stratification was based on local and international tools to monitor development and poverty and modified to be specific to COVID-19.^35,36^ The risk stratification of crowding considered that the household’s number of rooms also included shared living spaces. Households with multiple water sources were attributed the lowest possible risk.

**TABLE 1:** Risk stratification of the infrastructural determinants.

Infrastructural determinants	Risk		
	Low risk (LR)	Medium risk (MR)	High risk (HR)
Water source	Piped water inside the dwelling	Access to water on the property (e.g., piped water in the yard)	Access to water off the property
Toilet exposure	Private household toilet with associated handwashing facilities	Either a private household toilet with no handwashing facilities; or a shared toilet with other households with handwashing facilities	A shared toilet with other households without handwashing facilities
Indoor pollution	Household uses electricity, gas, or solar power	Household uses a combination of low- and high-risk sources	Household uses biomass fuels
Crowding (Household person-to-room ratio)	≤ 0.5	Between > 0.5 and ≤ 1	> 1

*Source*: Marcus SM, Marcus TS. Infrastructural inequality and household COVID-19 vulnerability in a South African urban settlement. J Urban Health. 2022;99:571–581. https://doi.org/10.1007/s11524-022-00625-7

### Household-head demographics

The demographics of interest were the age and gender of the household-head. Age was calculated from the date of birth as the expected age at the start of 2020, and households were divided into two group: those headed by people ≥ 65 years and < 65 years. Gender of the household-head was analysed as a dichotomous variable.

### Data analysis

Data were analysed using Stata 17^®^, with missing variables’ values imputed using multivariate imputation by chained equations with 20 iterations.^13^ Using the age groups and genders of the household-heads, the average number of people and rooms per dwelling was calculated. The ≥ 65 years headed households (HHs) group was further disaggregated by gender as, based on the literature, males ≥ 65 years HHs have the greatest risk of losing their household-head.^3^ The proportion of households by dwelling type was also calculated, with dwelling types being ‘formal house’, ‘room’, ‘collective living quarters’ (e.g., hostels and orphanages), ‘shack’ (an informal dwelling composed of makeshift materials such as corrugated iron), and ‘other’ (e.g., huts or tents).

Ordinal logistic regression was used to determine the odds of these households falling into HR categories for each of the four infrastructural determinants of COVID-19. Statistical significance was tested using Pearson’s chi-squared test with a cut off value of < 0.05. The proportion of HR categories based on the age and gender of the household-head was also calculated.

### Ethical considerations

This project is covered under the Researching the Development, Application, and Implementation of Community Oriented Primary Care (COPC) ethics protocols approved by the University of Pretoria Faculty of Health Science Research Ethics Committee (102/2011).

The secondary data analysed were deidentified and anonymised prior to the researchers gaining access.

## Results

Of the 13 985 households, the majority were headed by someone < 65 years (81.3%), and there were marginally more female-headed households (FHHs) (51.8%) than male-headed households (MHHs) (48.2%). The average number of people per household was 2.4 and the average rooms per household was 3.1. Table 2 depicts the average number of people and rooms per household by household-head demographics. On average, FHHs has more people (2.6) than MHHs (2.2) but also had slightly more rooms (3.2 vs. 2.9). The ≥ 65 years HHs had slightly more people on average (2.6) than < 65 years HHs (2.4) but had substantially more rooms (3.6 and 2.9, respectively). Female ≥ 65 years HHs had not only the highest average of people per household (2.8) but also the highest number of rooms per household (3.9) – both higher than male ≥ 65 years HHs (2.3 people and 3.3 rooms).

**TABLE 2:** Average number of people and rooms per household.

Household	Average number of people per household	Average number of rooms per households
Overall	2.4	3.1
FHHs	2.6	3.2
MHHs	2.2	2.9
< 65 years HHs	2.4	2.9
≥ 65 years HHs	2.6	3.6
Female > 65 years HHs	2.8	3.9
Male > 65 years HHs	2.3	3.3

FHHs, female-headed households; MHHs, male-headed households; HHs, headed households.

Table 3 shows the proportion of different dwelling types, with the most common dwelling type being shack (49%) followed by a house (37%) and room (10%). Female ≥ 65 years HHs had the greatest proportion of houses (64%), followed by male ≥ 65 years HHs (46%), and FHHs (44%). Both MHHs and < 65 years HHs had the highest proportion of shacks as a dwelling type (54%). This was followed by the FHHs (45%). Female ≥ 65 years HHs, however, had the lowest proportion of shacks (26%).

**TABLE 3:** Proportion of households living in different dwelling types.

Dwelling type	Group						
	Overall (%)	FHHs (%)	MHHs (%)	≥ 65 years HHs (%)	Female ≥ 65 years HHs (%)	Male ≥ 65 years HHs (%)	< 65 years HHs (%)
House	37	44	30	55	64	46	33
Room	10	9	10	9	7	11	9
CLQ	3	1	5	3	1	5	3
Shack	49	45	54	31	26	37	54
Other	1	1	1	2	1	2	1

**Total**	**100**	**100**	**100**	**100**	**100**	**100**	**100**

FHH, female-headed households; MHH, male-headed households; HHs, headed households; CLQ, collective living quarters.

From the ordinal logistic regression analysis (Table 4), the odds of falling in the HR category for all determinants was significantly lower for ≥ 65 years HHs, compared to < 65 years HHs. This was most pronounced for indoor pollution and crowding, with odds ratios for ≥ 65 years HHs being 0.42 (confidence interval [CI] = 0.39–0.46, *p* ≤ 0.001) and 0.52 (CI = 0.48–0.57, *p* ≤ 0.001), respectively. The odds of MHHs falling into the HR category compared to FHHs were greater for three of the infrastructural determinants, namely Water Source (OR = 1.32, CI = 1.23–1.41, *p* ≤ 0.001), Toilet Exposure (OR = 1.36, CI = 1.27–1.46, *p* ≤ 0.001), and Indoor Pollution (OR = 1.47, CI = 1.37–1.67, *p* < 0.001). However, for Crowding, MHHs’ odds of falling into the HR category were lower than FHHs’ (OR = 0.80, CI = 0.74–0.85, *p* ≤ 0.001).

**TABLE 4:** Odds of falling into the high-risk category for different households.

Infrastructural determinants	Risk category	Reference group	Household heads	Reference group	Odds ratios	*p*	95% CI
Water source	HR	MR + LR	≥ 65 years	< 65 years	0.64	0.000	0.58–0.70
			Male	Female	1.32	0.000	1.23–1.41
			Female ≥ 65 years	< 65 years	0.50	0.000	0.45–0.56
			Male ≥ 65 years	< 65 years	0.83	0.003	0.73–0.94
Toilet exposure	HR	MR + LR	≥ 65 years	< 65 years	0.77	0.000	0.70–0.84
			Male	Female	1.36	0.000	1.27–1.46
			Female ≥ 65 years	< 65 years	0.67	0.000	0.61–0.75
			Male ≥ 65 years	< 65 years	0.88	0.037	0.77–0.99
Indoor pollution	HR	MR + LR	≥ 65 years	< 65 years	0.42	0.000	0.39–0.46
			Male	Female	1.47	0.000	1.37–1.57
			Female ≥ 65 years	< 65 years	0.32	0.000	0.29–0.37
			Male ≥ 65 years	< 65 years	0.55	0.000	0.49–0.63
Crowding	HR	MR + LR	≥ 65 years	< 65 years	0.52	0.000	0.48–0.57
			Male	Female	0.80	0.000	0.74–0.85
			Female ≥ 65 years	< 65 years	0.55	0.000	0.50–0.62
			Male ≥ 65 years	< 65 years	0.50	0.000	0.44–0.57

HR, high-risk; MR, medium risk; LR, low risk; CI, confidence interval.

A breakdown of the ≥ 65 years HHs group by gender shows a similar pattern of infrastructural risk as above: the male ≥ 65 years HHs, however, only had marginally lower odds of falling into the HR category for crowding than female ≥ 65 years HHs.

**FIGURE 1:**
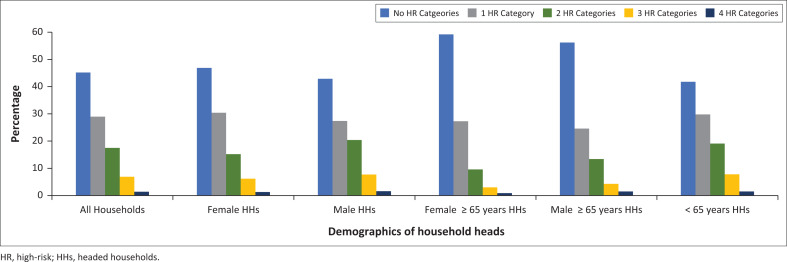
Proportion of high-risk categories by demographic groups.

Figure 1 demonstrates the proportion of HR categories among the age and gender groups. In all groups, there was a decrease in the proportion of households as the number of HR categories increased, with the highest percentage of households having no HR categories and the lowest percentage having all four. The rate of decline was the greatest for female ≥ 65 years HHs, with 59.2% having no HR categories and 0.9% having all four. Male ≥ 65 years HHs had the second highest proportion with no HR categories (56.2%) but also had one of the highest proportions with all four (1.5%). Male HHs and < 65 years HHs had the largest proportion of two, three, and four HR groups; however, FHHs had the highest proportion of one HR category, predominantly because of crowding.

## Discussion

The study aimed to investigate relative COVID-19 vulnerability in the township of Mamelodi through the analysis of the complex intersections between household infrastructure and the age and gender of the household-heads. As demonstrated by the results, varying degrees of relative advantage and disadvantage for COVID-19 vulnerability were found for each of these factors and at their intersections. In Mamelodi, ≥ 65 years HHs had a greater proportion of formal housing and were more likely to be in the lower risk categories for all four infrastructural determinants of COVID-19 compared to the < 65 years HHs. This is contrary to the narrative of uniform vulnerability among the elderly in LMICs.^37^

Despite ≥ 65 years HHs having an increased risk of losing their household-heads because of increased risk of fatal COVID-19, they have increased infrastructural protection in Mamelodi. The practical implication is that < 65 years HHs require more infrastructural support across all four infrastructural determinants. This should not, however, come at the expense of ≥ 65 years HHs as their resources result in greater protection for more community members as, on average, they have more people per household than < 65 years HHs.

When looking at gender, a greater proportion of MHHs live in informal housing compared to the FHHs and are more likely to fall into the HR categories for water source, toilet exposure, and indoor pollution, but not crowding. Even though a lower proportion of MHHs are formal with fewer rooms on average than FHHs, they have disproportionately fewer people, resulting in a lower risk and frequency of crowding. In this context, the infrastructural support required by MHHs should focus on WASH and energy source infrastructure as these are the areas that their households are most at risk of.

The greater infrastructural protection that FHHs have with respect to WASH infrastructure and indoor pollution have benefits for the wider community, as they have significantly more people, on average, living in their households. However, the gendered care load placed on these households creates its own COVID-19 risk through crowding. Given the high rate of spread among household members,^38^ crowding is a significant risk factor for both COVID-19 infection and severity.^39^ This highlights the need for infrastructural support for FHHs focusing on crowding relief measures in Mamelodi.

An interesting picture emerges when looking at the intersection between age and gender. Households headed by people ≥ 65 years have lower odds of falling in the HR categories for all four infrastructural determinants. Their disaggregation by gender, however, shows that female ≥ 65 years HHs are more likely to be in the lower risk categories for water source, toilet exposure, and indoor pollution than male ≥ 65 years HHs. Notably, the previous significant difference in the gendered odds of being HR for crowding has almost been completely erased. Despite female ≥ 65 years HHs having, on average, the highest number of people per household (2.8), they also have substantially more rooms per household than any other group (3.9), alleviating crowding generally seen with FHHs. While the increased number of people in these households is still likely to pose a risk through COVID-19 spread in household clusters, the lower levels of crowding along with better infrastructure in the other domains create a degree of relative privilege for female ≥ 65 years HHs.

This research also demonstrates an apparent gendered difference in care load. To illustrate, male ≥ 65 years HHs, have a high degree of relative advantage in multiple areas: they are less likely to be in the HR categories for all infrastructural determinants compared to < 65 years HHs; they have more rooms on average than FHHs (3.3 vs. 3.2) while having less people per household (2.3 vs. 2.6); and they have a higher proportion of formal housing compared to FHHs (46% compared to 44%). The greater space and formality seen in male ≥ 65 years HHs is thus not associated with a proportionate increase in household members, demonstrating that the high care load seen in FHHs is not merely a function of these factors. The benefits of improved infrastructure in FHHs are shared with more of the community and this is another factor that supports their prioritisation in the provision of state-subsidised housing.

Although age and gender impact on COVID-19 infrastructural vulnerability in various ways, there is household protection and vulnerability within all groups analysed. This is highlighted by male ≥ 65 years HHs who, despite their relative advantage in many areas, had one of the highest proportions of four HR categories (1.5%), higher than the overall population (1.4%). Across all demographic groups, the highest proportion of households had no HR categories, however, there were highly vulnerable households in every group with all four infrastructural determinants HR (Figure 1). These especially vulnerable households require extensive infrastructural support for COVID-19, which can be difficult if they are hidden in a group thought to have relative advantage.

The complexity of COVID-19 vulnerability in a single community is apparent from the results, with intersecting relative advantages and disadvantages. Studies from other LMICs have highlighted the unique patterns of COVID-19 vulnerability across and within nations, cities, and even subdistricts, requiring contextual responses.^40,41^ This contextual diversity has also been noticed in the age and gender related impacts of COVID-19, not only with respect to morbidity and mortality, but as they relate to many of its social determinants including economic outcomes, health decision making, interpersonal relationships, and labour practices.^42,43^ Together with this study, they emphasise the need for interventions driven by contextual analysis to have the greatest impact on local health outcomes.

The health significance of historical infrastructural decisions is also evident in the data. As demonstrated by Marcus and Marcus,^13^ formal housing, especially a house, can improve infrastructural protection against COVID-19. The focus on FHHs in the provision of RDP housing likely had a significant impact on their higher proportions of houses in this study, especially at the intersection with increased age. This in turn reduced some COVID-19 infrastructural vulnerability for a greater proportion of the population because of the care load carried by these households. The potential health implications of the gendered focus of RDP housing provision demonstrates how a focus on justice and equity in infrastructure policy can improve future health outcomes.

Finally, while this study has focused on COVID-19 in Mamelodi, the results hold significance for many other diseases and contexts. In addition, the risk of facing another COVID-19-like pandemic is expected to increase in the coming decades.^44^ This increases the importance of analysing social determinants in local contexts, and health-conscious investment in infrastructure at a household level, with justice and equity at the core of policy.

### Limitations

The choice of infrastructural determinants analysed were limited by the data, with other variables such as ventilation likely to be important. The data were also purposively sampled and self-reported, potentially resulting in sampling and self-report bias. Lastly, the data collected characterised gender as a dichotomous variable and was conflated with sex. This has the potential to marginalise households with non-binary gender-conforming household-heads, rendering their potential vulnerability invisible.

## Conclusion

While demographic and infrastructural determinants provide insight into COVID-19 vulnerability, it is at their contextual intersections that their impacts become significant. The results from our study demonstrate that targeted and contextually relevant intervention and policy based on the age and gender of the household-head would likely have a significant impact on COVID-19 vulnerability. It is still important, however, to identify households that do not follow typical vulnerability trends for the context, otherwise one risks overlooking these marginalised households. After all, the purpose of such contextually specific analysis is to avoid the advantage- or disadvantage-only homogenising paradigms of vulnerability. Additionally, historical equity-focused policy in housing, namely South Africa’s RDP, appears to have had a net positive impact in COVID-19 infrastructural protection for socially vulnerable groups, specifically households headed by women and the elderly. It also benefits the community at large because of the care load carried by these households. This protection is likely to be true for other diseases with transmission mechanisms or hygiene mitigation strategies similar to COVID-19.^45,46^ This study’s findings emphasise the importance of analysing contexts with an understanding of their social reproduction. This understanding, in turn, allows for the identification of social policies that have a positive health impact in communities, as well as potential areas for targeted interventions in current and future health crises.
